# Supplementary material for: Enhanced Wettability, Hardness, and Tunable Optical Properties of SiCxNy Coatings Formed by Reactive Magnetron Sputtering
Source: Materials (Basel). 2023 Feb 9;16(4):1467. doi: 10.3390/ma16041467 (PMC9961253; doi:10.3390/ma16041467)
Supplement: Supplementary file 1 [file materials-16-01467-s001.zip › materials-2130972-supplementary.pdf]

Supplementary Materials

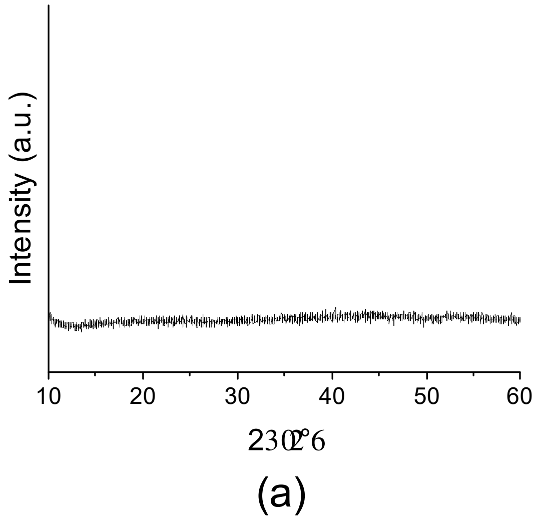

Figure S1. XRD patterns of SiC_xN_y films sputtered at (**a**) 25 and (**b**) 300 °C.

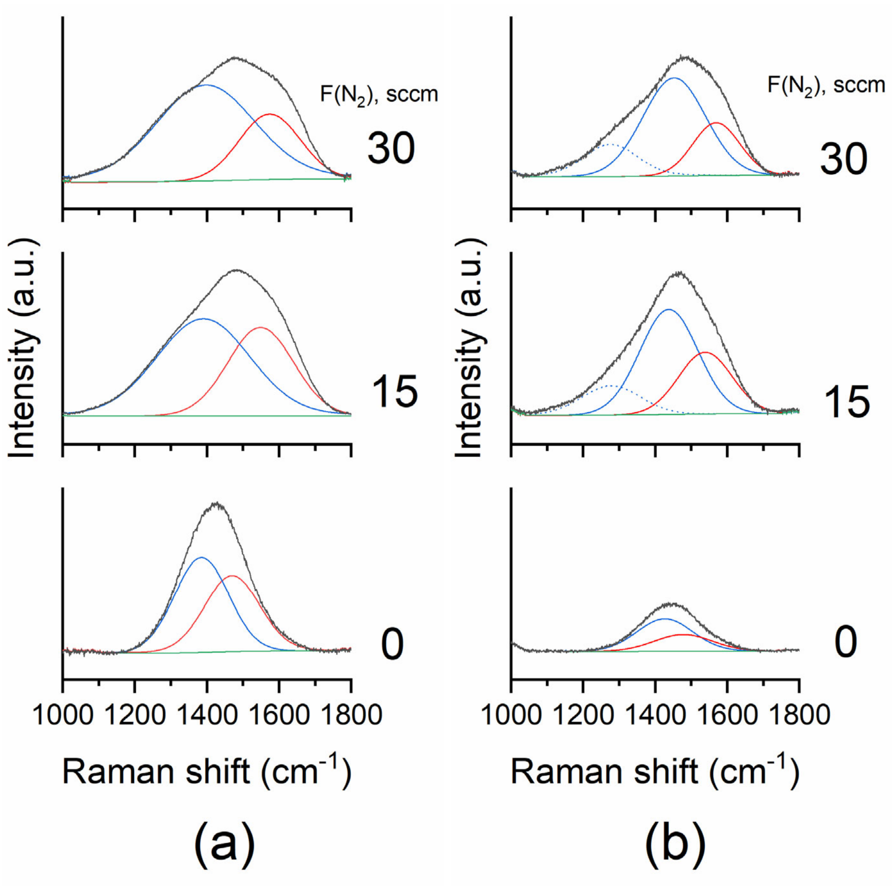

Figure S2. Deconvolution of Raman spectra of SiC_xN_y films sputtered at (**a**) 25 and (**b**) 300 °C.
